# Optimization of Porous Silicon Conditions for DNA-based Biosensing via Reflectometric Interference Spectroscopy

**DOI:** 10.22074/cellj.2019.5598

**Published:** 2018-08-05

**Authors:** Fereshteh Rahimi, Somayeh Fardindoost, Naser Ansari-Pour, Fatemeh Sepehri, Farideh Makiyan, Azizollah Shafiekhani, Ali Hossein Rezayan

**Affiliations:** 1 Division of Nanobiotechnoloy, Department of Life Science Engineering, Faculty of New Sciences and Technologies, University of Tehran, Tehran, Iran; 2 Department of Physics, Sharif University of Technology, Tehran, Iran; 3 Biotechnology Group, Department of Life Science Engineering, Faculty of New Sciences and Technologies, University of Tehran, Tehran, Iran; 4 Department of Physics, Alzahra University, Tehran, Iran; 5 School of Physics, Institute for Research in Fundamental Sciences, Tehran, Iran

**Keywords:** Biosensor, BRCA1 Gene, Nanochip Analytical Device

## Abstract

**Objective:**

Substantial effort has been put into designing DNA-based biosensors, which are commonly used to detect presence of known sequences including the quantification of gene expression. Porous silicon (PSi), as a nanostructured base, has been commonly used in the fabrication of optimally transducing biosensors. Given that the function of any PSi-based biosensor is highly dependent on its nanomorphology, we systematically optimized a PSi biosensor based on reflectometric interference spectroscopy (RIS) detecting the high penetrance breast cancer susceptibility gene, BRCA1.

**Materials and Methods:**

In this experimental study, PSi pore sizes on the PSi surface were controlled for optimum filling with DNA oligonucleotides and surface roughness was optimized for obtaining higher resolution RIS patterns. In addition, the influence of two different organic electrolyte mixtures on the formation and morphology of the pores, based on various current densities and etching times on doped p-type silicon, were examined. Moreover, we introduce two cleaning processes which can efficiently remove the undesirable outer parasitic layer created during PSi formation. Results of all the optimization steps were observed by field emission scanning electron microscopy (FE-SEM).

**Results:**

DNA sensing reached its optimum when PSi was formed in a two-step process in the ethanol electrolyte accompanied by removal of the parasitic layer in NaOH solution. These optimal conditions, which result in pore sizes of approximately 20 nm as well as a low surface roughness, provide a considerable RIS shift upon complementary sequence hybridization, suggesting efficient detectability.

**Conclusion:**

We demonstrate that the optimal conditions identified here makes PSi an attractive solid-phase DNA-based biosensing method and may be used to not only detect full complementary DNA sequences, but it may also be used for detecting point mutations such as single nucleotide substitutions and indels.

## Introduction

Porous silicon (PSi), a nanostructure of silicon material with a high surface-to-volume ratio, versatile surface chemistry, and adjustable morphology and pore diameters, has been commonly used in the medical and therapeutic fields especially in fabricating sensors and biosensors (1, 2). In biosensing applications, PSi can be used as a suitable transducer in combination with a variety of detection methods including those based on electrical, electrochemical, optical and thermal methods (3-5). 

Reflectometric interference spectroscopy (RIS) based on PSi, as a biosensor, was first introduced by Lin et al. (6). Since then, due to its applicability as a label- free biosensor, this method has received considerable attention (5, 7-17). Briefly, in this approach, light is shined on the PSi surface and the interference pattern of reflected beams from both the PSi surface and the PSi/Si bulk interface are detected. In their interference pattern, the wavelength of the peaks (λ) is determined by:

mλ=2nd

Where m is the spectral order of the fringe and 2nd is the effective optical thickness (EOT, twice of the product of the refractive index (n) and the thickness (d) of the layer (18, 19). Upon applying a solution containing biomolecules to the PSi layers, the pores of PSi are filled with such biomolecules, which in turn cause variation in the refractive index. Consequently, the observed shift in the interference pattern may be used as a robust signal for biosensing applications. However, for the RIS-PSi method to work efficiently, the two conditions should be satisfied. First, the pore sizes of PSi must be sufficiently large to allow biomolecules to penetrate the pores freely. However, pores larger than a certain limit decrease sensitivity due to the reduction in surface area. Therefore, PSi pore size optimization is essential in accurately sensing biomolecules. Secondly, the PSi surface roughness and the roughness of the PSi/Si bulk interface should be sufficiently low. If achieved, when the light hits the sample, the light scattering from the surface and the PSi/Si bulk interface is reduced. This increases the mirror reflection and interference pattern becomes significantly visible (20, 21). 

*BRCA1* is the most highly-penetrant breast cancer susceptibility gene in breast and ovarian cancer (22). Detection of *BRCA1* at both genomic and transcriptomic levels is useful in breast and ovarian cancer diagnosis and its screening may identify individuals with a high risk of cancer development (23).

In this work, a 30-nucleotide long probe of BRCA1 exonic sequence was used to optimize the PSi fabrication conditions for efficient DNA-based RIS biosensing. Given that each nucleotide is approximately 0.3 nm and the length of the used DNA sequence is thus 9nm, pore sizes of the PSi surface was set to approximately 20 nm for efficient detection.

## Materials and Methods

### Preparation of porous silicon 

In this experimental study, p-type silicon wafers with resistivity of 0.5 O cm and thickness of 800 µm were used in the (100) crystallographic orientation. Prior to the electrochemical process, a simple cleaning procedure was undertaken [by soap, deionized (DI) water and ethanol]. However, in some cases, after this process, samples were placed inside the electrolyte [consisting of a 35:50:15 mixture of 38-40 wt. % hydrofluoric acid (HF): 97 wt. % ethanol: DI water] for 15 seconds, then rinsed with ethanol. Afterwards, they were sonicated successively in chloroform, acetone and ethanol respectively for 10 minutes. 

After the cleaning process, silicon substrates were placed inside an electrochemical cell and the electrochemical process was undertaken by two types of novel electrolyte mixtures. The first electrolyte was a 1:10 (38-40 wt. % HF: 95 wt. % N) N dimethylformamide (DMF) solution and samples prepared with this are named hereafter as D-samples. The current density and etching time in preparing samples based on this electrolyte are given in Table 1. 

**Table 1:** The electrochemical etching conditions for D-samples


Etching time (s)	Current density (mA/cm^2^)	Sample name

1D	8.8	300
2D	3.5	300
3D	1.8	300
4D	0.9	300


The next electrolyte consisted of a 35:50:15 mixture of 38-40 wt. % HF: 97 wt. % ethanol: DI water and samples prepared with this solution are named hereafter as E-samples. The samples were treated under different electrochemical etching conditions with the electrochemical process being undertaken in two steps on two E-samples (Table 2). However, between these two steps, the samples were inserted into a 1 M NaOH solution containing 10% ethanol for 5 minutes and sonicated in methanol for 15 minutes to remove the porous layer, which was formed in the first step.

### Characterization of porous silicon

Field emission scanning electron microscopes (FESEM) of two different models (Hitachi S-4160, Japan and TESCAN, MIRA3, USA) were used to characterize the PSi surface. The diameters and distribution of the pores were determined by the ImageJ software (ImageJ, National Institutes of Health, Bethesda, Maryland, USA).

### Functionalization of porous silicon

#### Oxidization 

The HF reaction in the electrochemical process causes the prepared PSi surface to become extremely active with H-bond formations (e.g. Si-H, Si-H2 and Si-H3). These H-bonds are not stable in environmental conditions due to the exchange with oxygen groups, resulting in surface oxidation (24). To stabilize, as well as hydrophilize, the PSi surfaces, which is an essential criterion in biological applications, samples were exposed to hydrogen peroxide (35%, v/v, Merck) for 90 minutes in a dark place at room temperature (RT) (25, 26). The wafers were then rinsed with deionized water and subsequently dried. 

#### Silanization

To add the linkers, oxidized PSi was immersed in 3-amionpropyltriethoxysilane (APTES, 5% solution, Merck) in a water/methanol mixture (v/v=1:1) for 20 minutes at RT. PSi samples were rinsed with deionized water and then baked in the oven at 110°C for ten minutes to maximize crosslink between the functional groups. Next, the PSi samples were immersed in glutaraldehyde (GA, 2/5%, Merck), which was diluted in 20 mM HEPES buffer (pH=7.4), for 30 minutes and finally rinsed three times with deionized water to remove excess GA (27, 28).

### Immobilization of the DNA Probe

The DNA probe, a 30-nucleotide long probe of BRCA1 sequence (5´NH2-GAGCAAGAGAATCCCAGGACA GAAAGGTAA-3´; Macrogen, Korea) was immobilized by linkers on the surface of modified PSi. Briefly, 50 µM of the probe solution was placed on the surface. Samples were then incubated at 37°C for 2 hours. Finally, the prepared samples were rinsed with deionized water three times to remove excess and mobile DNA (27, 28). 

**Table 2:** The electrochemical etching conditions for E-samples


Sample name	First step		Second step	
	Current density (mA/cm^2^)	Etching time (s)	Current density (mA/cm^2^)	Etching time (s)

1E	17.6	300	-	-
2E	35.2	300	-	-
3E	70.5	300	-	-
3E^*a^	70.5	300	-	-
4E^b^	70.5	60	70.5	60
5E^b^	17.6	30	70.5	300


a; Additional cleaning procedure by the HF electrolyte and sonication in chloroform, acetone and ethanol, b; NaOH-treated between the two steps, and HF; Hydrofluoric acid.

### Detection

#### Hybridization of the Target DNA

The target DNA, a 30-nucleotide complementary sequence of *BRCA1* (5´-TTACCTTTCTGTCCTGGGATTCTCTTGCTC- 3´, Macrgoen, Korea) and thus complementary to the probe DNA, was allowed to hybridize on the surface of modified PSi with the probe DNA. Briefly, PSi samples were exposed to 50 µM of target DNA and incubated at 37°C for 20 minutes. After the incubation period, samples were rinsed with deionized water to remove unhybridized DNA.

#### Reflectometric interference spectroscopy

RIS was implemented by using a tungsten lamp illuminating the surface through an optical fibre. A collimator was then used to collect the reflected beam using an objective lens coupled with a multimode fibre which was directed into a spectrophotometer (EPP2000HR) with a spectral resolution of 0.5 nm. The EOT values were extracted from the RIS spectra. Briefly, the reflected spectrum was an interference pattern containing successive maxima and minima. According to equation (1), the wavelengths of two neighboring fringe maxima (λ_1_ and λ_2_,λ_1_ >λ_2_) are described by the following equations:

$m\lambda_{1}=\mathrm{2nd}$ and $(m+1)\lambda_{2}=\mathrm{2nd}$ (2)

where m and m+1 are the spectral order of these fringes respectively. Hence: 

$$m\lambda_{1}=(m+1)\lambda_{2}$$

By extracting the wavelengths of two arbitrary neighboring fringe maxima (λ_1_ and λ_2_) from any RIS spectrum, m and EOT (i.e. 2nd) can be calculated using equations (3) and (2) respectively (19, 29). 

## Results

We used two novel electrolyte media, DMF and ethanol, for RIS-PSi biosensing. PSi surface analysis and spectroscopic characterization of each PSi sample is presented separately below.

### D-samples 

In this section, we present the analysis of D-samples prepared in the DMF electrolyte.

#### Field emission scanning electron microscopy analysis

**Fig.1:**
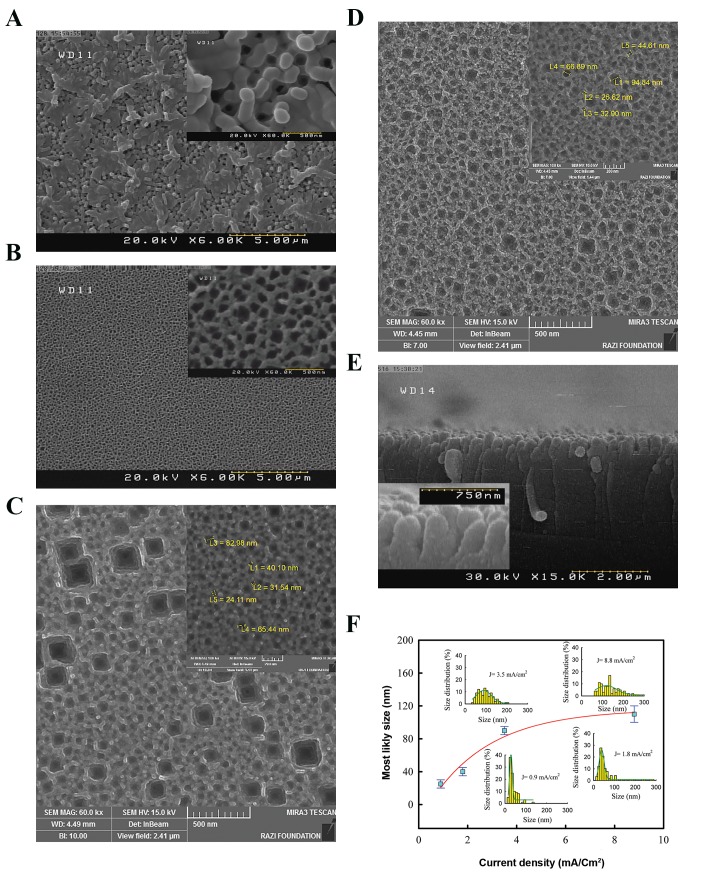
FE-SEM images of D samples and respective image analysis. A. Surface image of sample 1D, B. Surface image of sample 2D, C. Surface image of sample 3D, D. Surface image of sample 4D, E. Cross-section image of sample 2D (insets: high resolution images), and F. Modal pore size according to the magnitude of the current density in the electrochemical etching process (insets: the distribution of pore sizes for each current density).

Since surface roughness and distribution of pore diameters are important for biosensing detection, FESEM images of the samples were taken to assess the effect of electrolyte media and applied currents on these two factors. In sample 1D, based on a current density of 8.8 mA/cm^2^, large-diameter pores with sizes around 100 to 300 nm were formed (Fig .1A). Also, bigger cavities were observed of which some were distributed non-uniformly. It is possible that the walls between some cavities have been destroyed and thus resulting in larger merge cavities. The current density was then reduced to 3.5 mA/cm^2^ for sample 2D (Fig .1B). This reduced pore sizes to around 60-200 nm along with a uniform distribution. In sample 3D, we continued the optimization process by applying a lower current density at 1.8 mA/cm^2^. Smaller pores in the range of 20-100 nm were formed (Fig .1C), however, larger merged cavities of smaller cavities were still observed. By halving the current density to 0.9 mA/cm^2^ (sample 4D), the average size of pores was reduced to 50nm and a uniform distribution was obtained (Fig .1D). Images were then taken from the cross-section to analyse roughness of PSi samples (Fig .1E). Image analysis showed the formation of the rough surface at the interfaces of PSi/air and especially PSi/Si bulk of sample 2D where the upper surface of PSi is rather rough. However, due to the non-uniform growth of pores, depths of pores are completely unequal and consequently the interface of PSi/ bulk Si is extremely rough. This level of roughness in PSi samples which are anodized by the DMF electrolyte is in agreement with our previous study (30). ImageJ was used to quantify the size distribution of the pores according to the applied current densities (Fig .1F). The decrease in current density from 8.8 mA/cm^2^ to 0.9 mA/cm^2^ resulted D in a significant decrease in the modal pore size from 110 nm to 25 nm, which is consistent with others reports (7, 31). In addition, at a lower current density, a more uniform size distribution was observed. 

#### Reflectometric interference spectroscopy

RIS was implemented by a EPP2000-HR spectrometer. The obtained spectra were measured at wavelengths between 300 and 850 nm. At first, we measured the reflectance spectrum of the silicon substrate (Fig .S1) (See Supplementary Online Information at www.celljournal. org) which showed a peak around 370 nm as also reported by others (20, 32). The same measurement was undertaken for sample 2D and the same peak at the wavelength of 370 nm was observed (Fig .S1) (See Supplementary Online Information at www.celljournal.org) with no interference pattern. The reflectance spectra of the rest of the D-samples did not show any meaningful interference pattern. 

### E samples

In this section, we present the characterizations of E-samples prepared in the ethanol electrolyte.

### Field emission scanning electron microscopy

**Fig.2:**
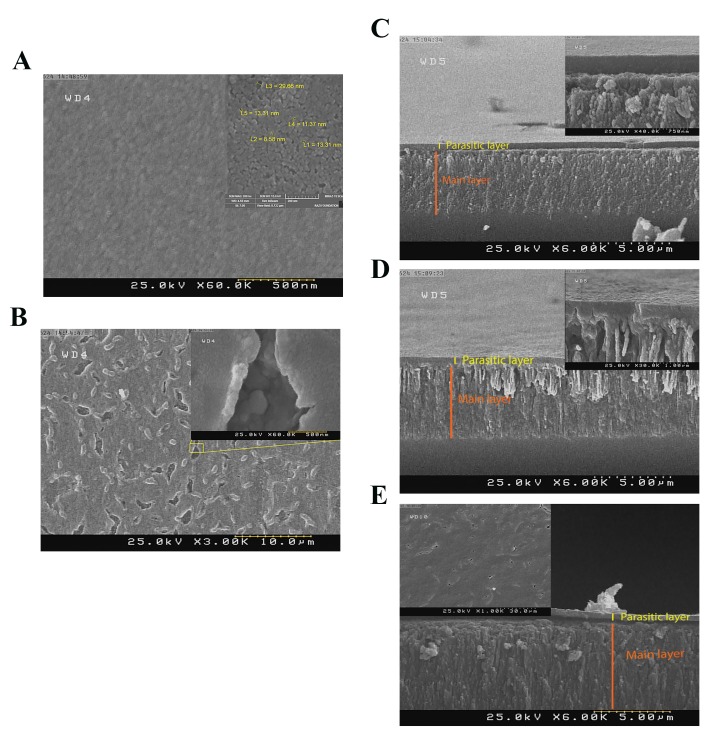
FE-SEM images of E samples. A. Surface image of sample 2E, B. Surface image of sample 3E, C. Cross section image of sample 2E, D. Cross section image of sample 3E (insets: high resolution images), and E. Cross section (main plot) and surface (inset) images of sample 3E*.

**Fig.3:**
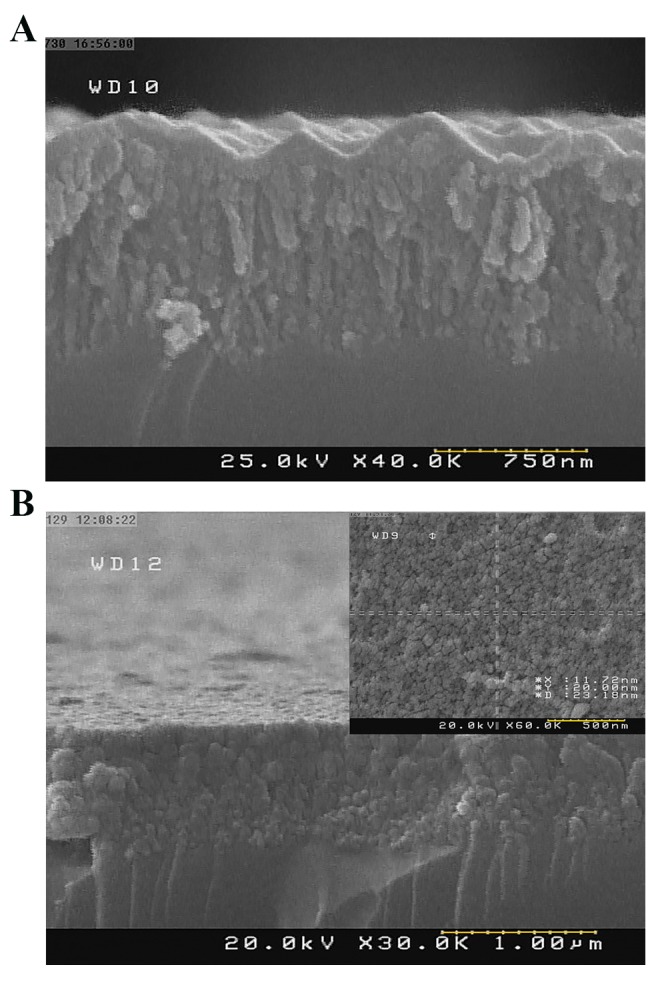
FE-SEM images of E samples with two-step electrochemical processing. A. Cross section image of sample 4E and B. Cross section image of sample 5E (inset: image of the surface of sample 5E).

FE-SEM images of sample 2E are illustrated at two magnifications in Figure 2A. Uniform surface with very small pores of about 5 to 30 nm was formed on the surface. FE-SEM image of sample 1E did not show any observable difference in porosity compared with sample 2E. Next, by increasing the current density in sample 3E, the outermost surface showed no change in porosity (compare the main plot of Fig.2A and the inset plot of Fig.2B). In addition, D increasing current density can cause the appearance of scab on the surface. By going inside the scab, the SEM images showed that the underneath layer has a different porosity (Fig .2B). Also, images taken from the cross-section of samples 2E (Fig .2C) and 3E (Fig .2D) showed the presence of a “crust” layer on the top of the porous layer. Image analysis showed that the thickness of both parasitic and main layers were grown by increasing the current density (Fig .S2) (See Supplementary Online Information at www.celljournal. org). Scanning electron micrograph of sample 3E* (Fig .2E) showed that the applied additional cleaning process was not successful in preventing the formation of a parasitic layer. However, in these samples, formation of cracks is more dominant (compare insets of Fig.2B and Fig.2E).

Formation of the porous layer by a two-step electrochemicalprocess was then investigated by FE-SEM (Fig .3). The cross- section of sample 4E exhibited high roughness, indicatingthat the applied first step electrochemical etching did notalter the roughness of the PSi/Si bulk (Fig .3A). Thus, afterNaOH treatment, which removes PSi layers (both of parasitic and main layers), the silicon surface would remain rough. Therefore, in the second step electrochemical etching, pore formation was initiated based on the rough background. However, by decreasing the current density and the time of etching in the first step, suitable pore diameters were grown on the surface with no parasitic layer (Fig .3B).

### Reflectometric interference spectroscopy

**Fig.4:**
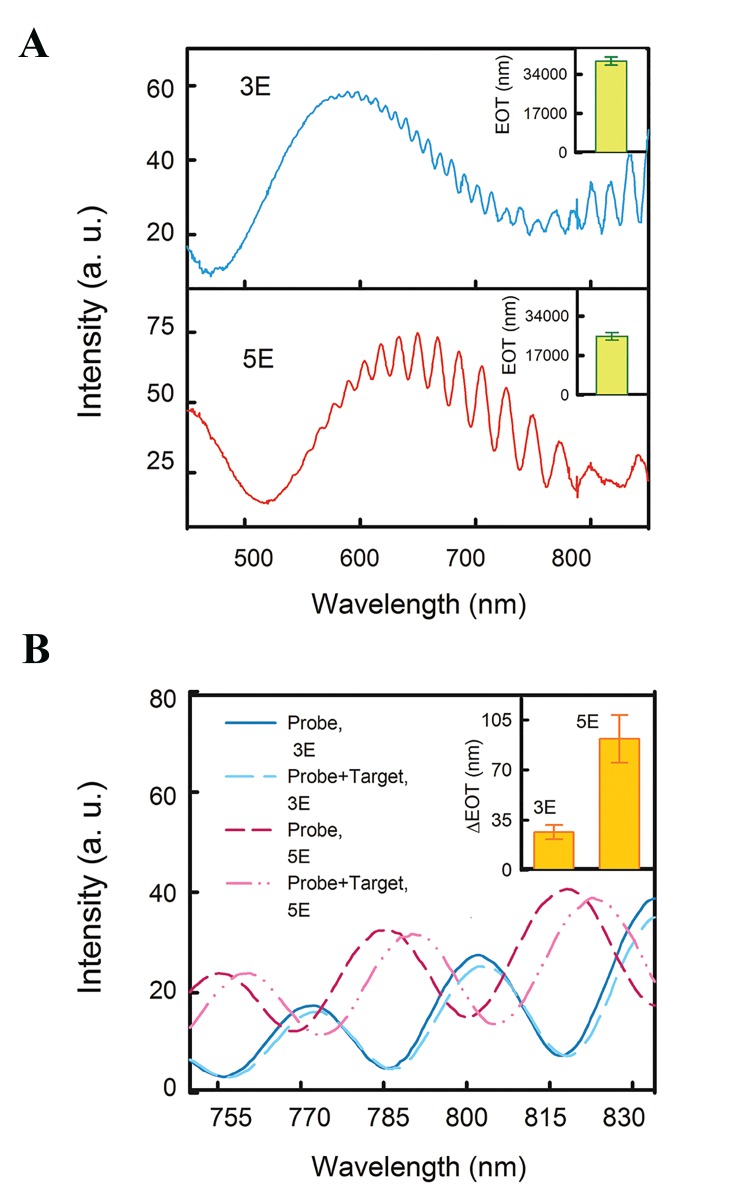
The reflectance spectra analysis of E samples for DNA detection. A. fresh 3E and fresh 5E samples (insets: EOT extracted from the spectra) and B. samples 3E and 5E before and after the hybridization of the DNA target (inset: change in EOT of samples 3E and 5E due to the hybridization of target DNA molecules).

RIS was undertaken for wavelengths between 300-850 nm. The 4E sample did not show any interference pattern. RIS was then implemented for 3E and 5E samples. The reflectometry measurement based on 3E and 5E samples showed both interference pattern (Fig .4A) as well as silicon reflection peaks at about 370 nm. Extracted EOT from RIS spectra for 3E and 5E samples (three samples for each case) are shown in the insets of Figure 4A. Since EOT is equal to 2nl [twice of the product of the refractive index (n) and the thickness (d)], any change in porosity (and consequently refractive index) or thickness alter the EOT value. Thus, the reproducibility of extracted EOT was used as representative of the reproducibility of these samples. Although FE-SEM images of replicate samples could have been used to check reproducibility, the EOT method was not only more cost-effective and convenient, but it was also readily quantifiable. After each functionalization step, these characteristics (interference pattern) remained which is illustrated between 750-835 nm for the 3E and 5E samples after immobilization of the DNA probe in Figure 4B. After hybridization of the target DNA, the interference patterns of both 3E and 5E samples were shifted to the larger wavelengths (i.e. red shift). However, the red shift for the 5E sample was larger than the 3E sample. The change in EOT of each sample is depicted in the inset of Figure 4B. We used non-complementary target DNAs for both samples 3E and 5E after probe immobilisation but did not observe any variation in the extracted EOT (data not shown), suggesting that the extracted EOT are true representatives of the interference signal from the hybridization of complementary target probes.

## Discussion

For the RIS method to work efficiently as a new transducer in biosensing on PSi substrates, two conditions should be met. Firstly, PSi surface roughness and the roughness of the PSi/Si bulk interface should be sufficiently low. In general, the specular reflectance at normal incidence is given by equation 2 (21, 33, 34): 

$$R_{s}=R_{0}\exp[-\frac{{\mathrm{(4\pi \sigma )}}^{2}}{\lambda^{2}}]$$

where R_S_, R_0_, σ and λ are the specular reflectance of the rough surface, specular reflectance of a perfectly smooth surface of the same material, the roughness of the surface and the wavelength of the incident light respectively. Based on equation 2, the specular reflectance is inversely proportional to the roughness of the surface. In the case of PSi, the reflectance spectra include the scattering from the volume or the inner surface and, the scattering from the air/PSi interface and the scattering from the PSi/bulk Si. It has been shown that the PSi/Si bulk scattering plays the most dominant role (21, 33, 35). We therefore suggest that the un-meaningful interference patterns of D-samples and sample 4E are due to their high surface roughness on the surface and especially at the PSi/ bulk Si interface (Fig .S3) (See Supplementary Online Information at www. celljournal.org).

Secondly, the pore sizes of PSi must be optimum. Because of the reduction in surface area and consequently reduction in sensitivity, large pores are not desirable. Small pores which do not permit biomolecules to penetrate the pores freely are also not useful. The pore sizes could be easily controlled by adjusting the current density in the electrochemical process in D samples. However, the high surface roughness of these samples is an obstacle. On the other hand, the formation of the crust layer in E samples which are reported previously (19, 31, 32, 36-40) is also a serious problem. This crust layer plays as a parasitic layer and can prohibit the diffusion of biomolecules into the main layer. Therefore, applying some removal techniques were one of the challenges in our work which is also previously studied and reported for different techniques by others (19, 32, 39, 40). Finally, by using a controlled two-step process in the ethanol electrolyte accompanied by removal of the parasitic layer in NaOH solution, we were able to satisfy the two essential conditions for PSi substrates. As a consequence, DNA sensing reached its optimum with these samples, where pore sizes were approximately 20 nm wide and surface roughness was as low as possible. Upon complementary sequence hybridization, we observed a considerable RIS shift, suggesting efficient detectability of DNA molecules.

## Conclusion

We introduce an optimization approach for controlling pore sizes on the PSi surface, optimum filling with short DNA molecules and minimising PSi surface roughness to obtain superior biosensing performance based on RIS. Furthermore, the formation of the parasitic outer layer with high roughness created on top of the PSi prevents any RIS shifts and must be avoided by either of the two cleaning processes introduced. Finally, we demonstrate that the optimal conditions to obtain a considerable shift in RIS after complementary sequence hybridization are i. PSi formation in ethanol electrolyte in a two-step process and ii. Removal of the parasitic layer in NaOH solution. These optimal conditions makes PSi an attractive label-free method for DNA-based biosensing and may potentially be used to detect single nucleotide variants such as substitutions and indels. Nevertheless, to validate this PSi-based biosensor, it would be essential to identify the detection limit and the dynamic response range of this chip, which we aim to undertake as part of the follow-up of this study. 

## Supplementary PDF



## References

[1] Canham LT (1997). Properties of porous silicon.London: INSPEC.

[2] Santos HA (2014). Porous silicon for biomedical application.Cambridge: Woodhead Publishing.

[3] Harraz FA (2014). Porous silicon chemical sensors and biosensors: a review. Sensors and Actuators B: Chemical.

[4] Dhanekar S, Jain S (2013). Porous silicon biosensor: Current status. Biosens Bioelectron.

[5] Jane A, Dronov R, Hodges A, Voelcker NH (2009). Porous silicon biosensors on the advance. Trends Biotechnol.

[6] Lin VS, Motesharei K, Dancil KP, Sailor MJ, Ghadiri MR (1997). A porous silicon-based optical interferometric biosensor. Science.

[7] Janshoff A, Dancil KPS, Steinem C, Greiner DP, Lin VS, Gurtner C (1998). Macroporous p-type silicon fabry-perot layers fabrication, characterization, and applications in Biosensing. J Am Chem Soc.

[8] Collins BE, Dancil KPS, Abbi G, Sailor MJ (2002). Determining protein size using an Electrochemically Machined pore gradient in silicon. Advanced Functional Materials.

[9] Steinem C, Janshoff A, Lin VS, Völcker NH, Ghadiri RM (2016). DNA hybridization- enhanced porous silicon corrosion: mechanistic investigations and prospect for optical interferometric biosensing. Tetrahedron.

[10] Pacholski C, Sartor M, Sailor MJ, Cunin F, Miskelly GM (2005). Biosensing using porous silicon double-layer interferometers: reflective interferometric fourier transform spectroscopy. J Am Chem Soc.

[11] Francia GD, Ferrara VL, Manzo S, Chiavarini S (2005). Towards a labelfree optical porous silicon DNA sensor. Biosens Bioelectron.

[12] Ouyang H, Striemer CC, Fauchet PM (2006). Quantitative analysis of the sensitivity of porous silicon optical biosensors. Appl Phys Lett.

[13] Schwartz MP, Alvarez SD, Sailor MJ (2007). Porous SiO2 Interferometric Biosensor for Quantitative Determination of Protein Interactions: Binding of Protein A to Immunoglobulins Derived from Different Species. Anal Chem.

[14] Rong G, Ryckman JD, Mernaugh RL, Weiss SM (2008). Label-free porous silicon membrane waveguide for DNA sensing. Appl Phys Lett.

[15] Rea I, Lamberti A, Rendina I, Coppola G, Gioffrè M, Iodice M (2010). Fabrication and characterization of a porous silicon based microarray for label-free optical monitoring of biomolecular interactions. J Appl Phys.

[16] Feng J, Zhao W, Su B, Wu J (2011). A label-free optical sensor based on nanoporous gold arrays for the detection of oligodeoxynucleotides. Biosens Bioelectron.

[17] Baranowska M, Slota AJ, Eravuchira PJ, Alba M, Formentin P, Pallarès J (2015). Protein attachment to silane-functionalized porous silicon: A comparison of electrostatic and covalent attachment. J Colloid Interface Sci.

[18] Hecht E (1998). Optics.

[19] Sailor MJ (2012). Porous silicon in practice preparation characterization and applications.

[20] Theiß W (1997). Optical properties of porous silicon. Surface Science Reports.

[21] Canham LT (1997). Properties of porous silicon.London: INSPEC.

[22] Miki Y, Swensen J, Shattuck-Eidens D, Futreal PA, Harshman K, Tavtigian S (1994). A strong candidate for the breast and ovarian cancer susceptibility gene BRCA1. Science.

[23] Culha M, Stokes D, Allain LR, Vo-Dinh T (2003). Surface-enhanced raman scattering substrate based on a self-assembled monolayer for use in gene diagnostics. Anal Chem.

[24] Canham LT (1997). Properties of porous silicon.London: INSPEC.

[25] Naveas N, Costa VT, Gallach D, Hernandez-Montelongo J, Palma RJ, Garcia-Ruiz JP (2012). Chemical stabilization of porous silicon for enhanced biofunctionalization with immunoglobulin. Sci Technol Adv Mater.

[26] Naveas N, Hernandez-Montelongo J, Pulido R, Torres-Costa V, Villanueva- Guerrero R, Predestinación García Ruiz J (2014). Fabrication and characterization of a chemically oxidized-nanostructured porous silicon based biosensor implementing orienting protein A. Colloids and Surfaces B: Biointerfaces.

[27] Zhang H, Jia Z, Lv X, Zhou J, Chen L, Liu R (2013). Porous silicon optical microcavity biosensor on silicon-on-insulator wafer for sensitive DNA detection. Biosens Bioelectron.

[28] De Tommasi E, De Stefano L, Rea I, Di Sarno V, Rotiroti L, Arcari P (2008). Porous silicon based resonant mirrors for biochemical sensing. Sensors (Basel).

[29] Huang K, Li Y, Wu Z, Li C, Lai H, Kang J (2011). Asymmetric light reflectance effect in AAO on glass. Opt Express.

[30] Razi F, Zad AI, Rahimi F (2010). Investigation of hydrogen sensing properties and aging effects of Schottky like Pd/porous Si. Sensors and Actuators B: Chemical.

[31] Dubin VM (1992). Formation mechanism of porous silicon layers obtained by anodization of monocrystalline n-type silicon in HF solutions. Surface Science.

[32] Chamard V, Dolino G, Muller F (1998). Origin of a parasitic surface film on p+ type porous silicon. J Appl Phys.

[33] Lérondel G, Romestain R, Barret S (1997). Roughness of the porous silicon dissolution interface. J Appl Phys.

[34] Bennett HE, Porteus JO (1961). Relation between Surface Roughness and Specular Reflectance at Normal Incidence. J Opt Soc Am.

[35] Lérondel G, Romestain R, Madéore F, Muller F (1996). Light scattering from porous silicon. Thin Solid Films.

[36] Watanabe Y, Arita Y, Yokoyama T, Igarashi Y (1975). Formation and properties of porous silicon and its application. J Electrochem Soc.

[37] Unagami T (1980). Formation mechanism of porous silicon layer by anodization in HF solution. J Electrochem Soc.

[38] Lust S, Lévy-Clément C (2002). Chemical Limitations of Macropore Formation on Medium-Doped p-Type Silicon. J Electrochem Soc.

[39] Errien N, Vellutini L, Louarn G, Froyer G (2007). Surface characterization of porous silicon after pore opening processes inducing chemical modifications. Appl Surf Sci.

[40] Sciacca B, Secret E, Pace S, Gonzalez P, Geobaldo F, Quignard F (2011). Chitosan-functionalized porous silicon optical transducer for the detection of carboxylic acid-containing drugs in water. J Mater Chem.

