# Supplementary material for: Optimization of Porous Silicon Conditions for DNA-based Biosensing via Reflectometric Interference Spectroscopy
Source: Cell J. 2018 Aug 5;20(4):584–91. doi: 10.22074/cellj.2019.5598 (PMC6099142; doi:10.22074/cellj.2019.5598)
Supplement: Supplementary file 1 [file Cell-J-20-584-s01.pdf]

www.celljournal.org

Supplementary Information for

Optimization of Porous Silicon Conditions for DNA-Based Bio-sensing via Reflectometric Interference Spectroscopy

Fereshteh Rahimi, Ph.D.[1]*, Somayeh Fardindoost, Ph.D.[2], Naser Ansari-Pour, Ph.D.[3]*, Fatemeh Sepehri, M.Sc.[1], Farideh Makiyan, M.Sc.[1], Azizollah Shafikhani, Ph.D.[4, 5], Ali Hossein Rezayan, Ph.D.[1]

1. Division of Nanobiotechnoloy, Department of Life Science Engineering, Faculty of New Sciences and Technologies, University of Tehran, Tehran, Iran
2. Department of Physics, Sharif University of Technology, Tehran, Iran
3. Biotechnology Group, Department of Life Science Engineering, Faculty of New Sciences and Technologies, University of Tehran, Tehran, Iran
4. Department of Physics, Alzahra University, Tehran, Iran
5. School of Physics, Institute for Research in Fundamental Sciences, Tehran, Iran

**Corresponding Address: Addresses: Division of Nanobiotechnoloy, Department of Life Science Engineering, Faculty of New Sciences and Technologies, University of Tehran, Tehran, Iran*
P.O.Box: 1439957131, Biotechnology Group, Department of Life Science Engineering, Faculty of New Sciences and Technologies, University of Tehran, Tehran, Iran
Emails: rahimi.f@ut.ac.ir , n.ansaripour@ut.ac.ir

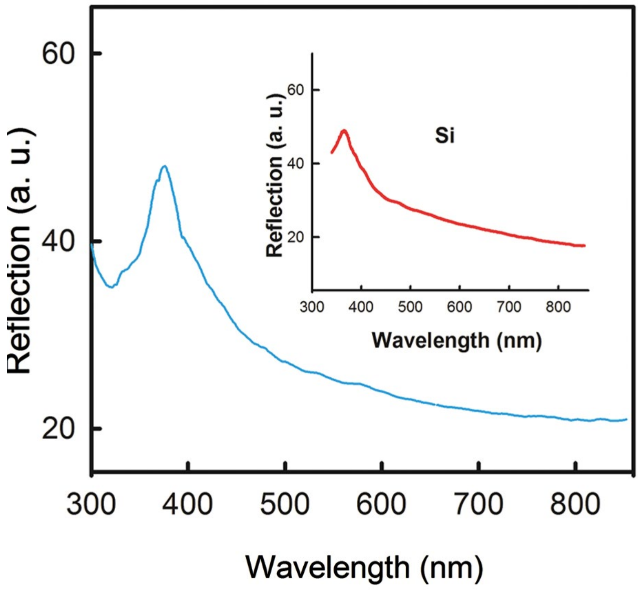

Fig.S1: Reflectance spectrum of sample 2D (inset: the reflectance spectrum of the silicon substrate).

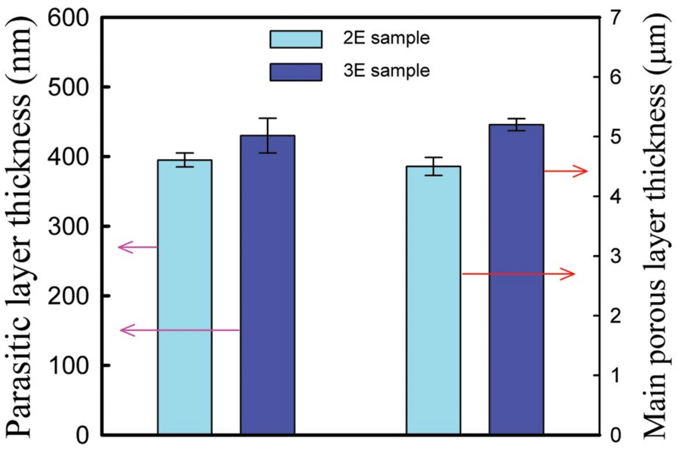

Fig.S2: Quantification of the thickness of the parasitic and main layers in samples 2E and 3E.

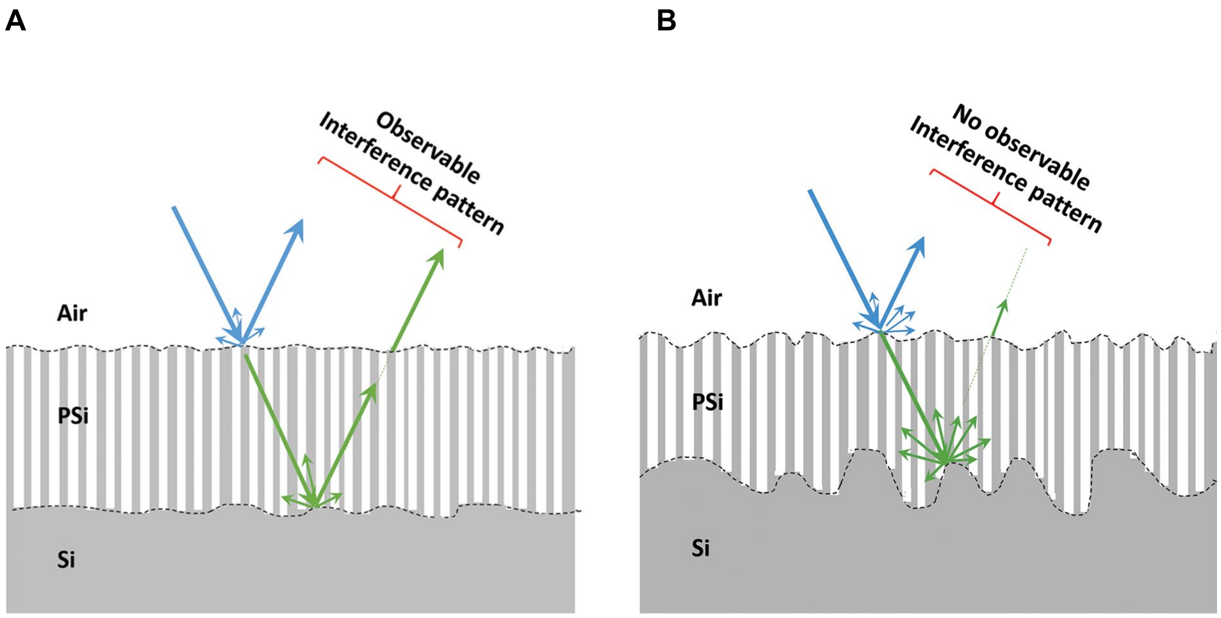

Fig.S3: The effect of surface roughness on reflection from the PSi thin film. **A.** small interface roughness and **B.** large interface roughness.
